# Pulsed-Mode Magnetic Field Measurements with a Single Stretched Wire System

**DOI:** 10.3390/s24144610

**Published:** 2024-07-16

**Authors:** Joseph Vella Wallbank, Marco Buzio, Alessandro Parrella, Carlo Petrone, Nicholas Sammut

**Affiliations:** 1 Department of Microelectronics and Nanoelectronics, Universita ta’ Malta, MSD 2080 Msida, Malta; 2 Technology Department, CERN—European Organization for Nuclear Research, 1217 Geneva, Switzerland; marco.buzio@cern.ch (M.B.); carlo.petrone@cern.ch (C.P.)

**Keywords:** single stretched wire, magnetic field measurements, particle accelerators, bending magnet, induction coil calibration

## Abstract

In synchrotrons, accurate knowledge of the magnetic field generated by bending dipole magnets is essential to ensure beam stability. Measurement campaigns are necessary to characterize the field. The choice of the measurement method for such campaigns is determined by the combination of magnet dimensions and operating conditions and typically require a trade-off between accuracy and versatility. The single stretched wire (SSW) is a well-known, polyvalent method to measure the integral field of magnets having a wide range of geometries. It, however, requires steady-state excitation. This work presents a novel implementation of this method called pulsed SSW, which allows the system to measure rapidly time-varying magnetic fields, as is often needed, to save power or gain beam time. We first introduce the measurement principle of the pulsed SSW, followed by a combined strategy to calculate the absolute magnetic field by incorporating the classic DC SSW method. Using a bending magnet from the Proton Synchrotron Booster located at the European Organization for Nuclear Research as a case study, we validate the pulsed SSW method and compare its dynamic measurement capabilities to a fixed induction coil, showing thereby how the coil calibration must be adjusted according to the field level. Finally, we assess the method’s measurement accuracy using the standard SSW as a reference and present an analysis of the primary noise contributors.

## 1. Introduction

**Figure 1:**
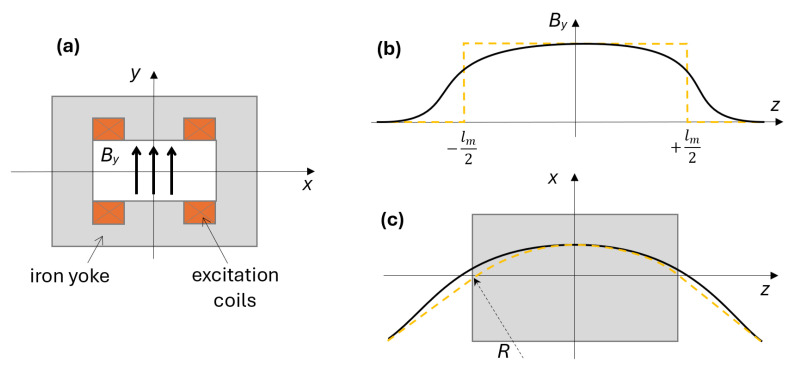
Schematic representation of the beam path in a dipole magnet, assuming small bending angle (i.e., $l_{m}\ll R$) so that the arc length of the path can be replaced by its projection onto the longitudinal axis *z*. Dashed curves refer to the hard-edge model. (**a**) Vertical cross-section of the magnet; (**b**) longitudinal field profile; (**c**) beam path (top view).

In synchrotrons, large sets of dipole electromagnets are used to bend the particle beam in the horizontal plane along a ring-shaped trajectory, according to the Lorentz force generated by a vertical magnetic field [1]. Over a magnetic cycle, the dipole field changes proportionately to the beam energy to ensure that the beam remains centered within the vacuum chamber. We consider a single bending magnet, as represented schematically in Figure 1, where the beam circulates along the longitudinal axis of the magnet *z*, and *x* and *y* represent the transverse and vertical directions, respectively. The vertical field component:

(1) $$B_{y}\left(x,y,z,I\left(t\right)\right)$$

is a function of the position and magnet excitation current I(t). The magnetic field is approximately proportional to the current, which is typically very uniform in the gap between the magnet poles and rolls off to zero rapidly in the surrounding fringe region. The main property of interest for synchrotron operation is the longitudinal integral of the field or, equivalently, its average $\bar{B}$:

(2) $$\bar{B}\left(I\left(t\right)\right)=\frac{1}{l_{m}}\int_{-\infty }^{+\infty }B_{y}\left(0,0,z,I\left(t\right)\right)\;dz$$

where $l_{m}$ is a reference length, defined as the length of the circular arc traced by the beam inside the magnet, under the assumption of constant field (hard-edge model, [1]):

(3) $$l_{m}=\frac{2\pi R}{N_{B}}$$

where *R* is the bending radius of the synchrotron and $N_{B}$ is the number of bending magnets powered in series. The reference length is usually close, but not necessarily identical, to the length of the magnet’s iron yoke. We also note that the total length of the accelerator ring is always greater than 2πR, because the beam trajectory between dipoles is generally composed of additional straight sections corresponding to different kinds of components, such as focusing quadrupoles or RF cavities. In practice, integration in (2) can be truncated to a short distance from both ends of the iron yoke, with 2.5 times the height of the magnet aperture being more than sufficient [2].

To program the current excitation cycles, machine operators need knowledge of the current-to-field relationship (2) with a relative precision of $10^{-4}$. However, the magnet response is usually affected (up to several percent) by nonlinear phenomena such as magnetic saturation, hysteresis, and eddy currents, which cannot easily be predicted by means of FE models or measured with beam-based techniques during operation. The most accurate and cost-effective approach is to measure the integral of the magnetic field directly using a reference magnet. Here, the two most widely used measurement methods are based on induction coils and single stretched wire (SSW) systems, which have the added benefit of providing information on the quality and uniformity of the main field component [3].

Induction coils represent one of the most basic types of magnetic field sensors. A coil typically consists of multiple loops of conducting wire, wound around a rectangular core, and positioned along the longitudinal axis of a magnet’s aperture [4]. The output voltage is proportional to the number of turns and the rate of change of the linked magnetic flux, according to Faraday’s law. A fixed coil is therefore suitable for measuring time-varying magnetic fields, in which case, however, it can register only their variation. Conversely, a coil rotating or translating in a stationary field coil can be used for absolute measurements but at the cost of a more complex mechanical setup. In both cases, very-high-quality results are possible, provided that the coil is adapted to match a magnet’s specific geometry. Unfortunately, manufacturing uncertainties increase with the length and number of turns, making accurate calibration of integral coils more difficult, as is discussed in Section 2.4.

With respect to induction coils, the single stretched wire (SSW) method presents complementary advantages and drawbacks. In a dipole magnet, the classic static SSW (s-SSW) method is used to measure the absolute integral of the magnetic field by displacing a single taut conducting wire perpendicularly to the field direction. A key advantage of this method lies in the flexibility to cover a wide range of magnet lengths and aperture widths with the same hardware. Moreover, the width of the flux linkage area swept by the stretched wire is defined with exquisite precision by the translation stages, which is often better than the micrometer level and in stark contrast with typical induction coils. As a result, the static SSW method is often used as a reference to calibrate the width of integral induction coils, as discussed in the next section. The limitations of this method are twofold; First, the setup can only measure steady-state magnetic fields. Secondly, as the method is equivalent to using a single-turn variable-geometry coil, the output voltage depends mainly on the magnetic field strength [5]. Other SSW methods exist where the magnet or wire is excited using an AC waveform. However, we shall not cover them here as they are mainly used to determine the magnetic axis of quadrupole magnets. Overall, both measurement methods are induction-based and, therefore, inherently linear to the field level, which is a key advantage over other types of magnetic sensors, such as Hall probes [6].

**Figure 2:**
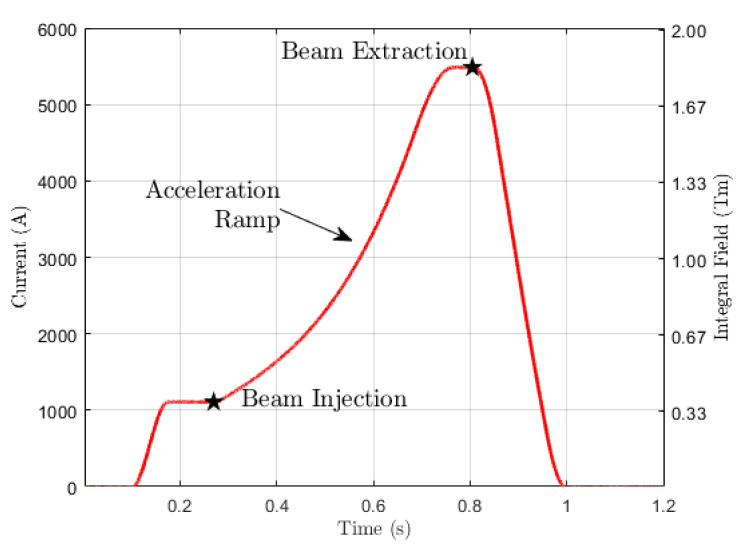
Current waveform powering the PSB bending magnets during a 2 GeV magnetic cycle.

In the following, we shall compare the performance of these measurement methods by taking a bending magnet currently in operation at the European Organization for Nuclear Research (CERN) as a case study. At CERN, the Large Hadron Collider (LHC) proton injector chain includes four synchrotrons that progressively increase the beam energy from 160 MeV to 6.5 TeV, thus limiting nonlinear effects that grow exponentially with the dynamic range of each ring. We focus on the first synchrotron in the chain, the Proton Synchrotron Booster (PSB), which accelerates protons up to 2 GeV. This synchrotron consists of $N_{b}=32$ dipoles that bend the beam with a radius R=8.3m, corresponding to a reference magnetic length of $l_{m}=1.630\;m$, as per Equation (3). The PSB bending dipoles are excited by the 1.2 s long current cycle depicted in Figure 2, which reaches a current level $I_{\max}=5400\;A$ at the extraction flat-top [7]. Here, the magnets produce an integrated field of $l_{m}{\bar{B}}_{\max}=1.835\;\mathrm{Tm}$, which we will use throughout the following analysis as a normalization reference. The magnetic cycle is characterized by a high ramp rate, with a ramp-up time of around 0.5 s that peaks at about 5 T/s. This rapid cycling allows for large numbers of protons to be accelerated while limiting thermal dissipation in the magnet’s excitation coils, which were originally designed to operate at the much lower level of ∼2300 A [8]. Unfortunately, high ramp rates induce substantial eddy currents in the iron yoke of an accelerator magnet, degrading the field uniformity and causing the magnetic field to lag behind the current waveform [9]. In the PSB, the eddy currents delay the magnetic field’s response by as much as 0.7 ms at beam extraction, corresponding to a field error of about 0.2%. This highly dynamic nature of the magnetic field compounds the need for accurate measurements while restricting the usage of the static SSW, thereby making the precise calibration of induction coils more difficult.

For this reason, we have developed the so-called pulsed SSW (p-SSW) method; a novel implementation of the SSW setup that enables the system to measure fast time-varying magnetic fields by combining dynamic measurements taken at different wire positions. In the context of our case study, the pulsed SSW setup serves mainly as a method to complement and cross-calibrate fixed coil measurements. The availability of suitable induction coils, especially with lengths on the order of meters, is limited by the lack of commercial offers and the level of resources needed to design, manufacture, and calibrate them. Due to its inherent flexibility in adapting to a wide range of geometries, the pulsed SSW method represents a very effective alternative.

This paper is structured as follows: In Section 2, we first introduce the operating principles of both the induction coil and the static and pulsed SSW implementations; then, in Section 3, we detail the test setup and measurement procedure of the PSB bending dipole. In Section 4, we present, analyze, and compare the results obtained with all three methods, highlighting how the pulsed SSW method has been used to calibrate a fixed induction coil. In Section 5, we make concluding remarks and outline planned developments.

## 2. Measurement Principle

In this section, we describe the measurement principle of the three methods used in this work. The symbol Φ shall denote absolute flux linked through an induction loop, whether an induction coil or a wire. The symbol ΔΦ shall be used for space or time flux differences (or, equivalently, excitation current).

### 2.1. Static SSW

**Figure 3:**
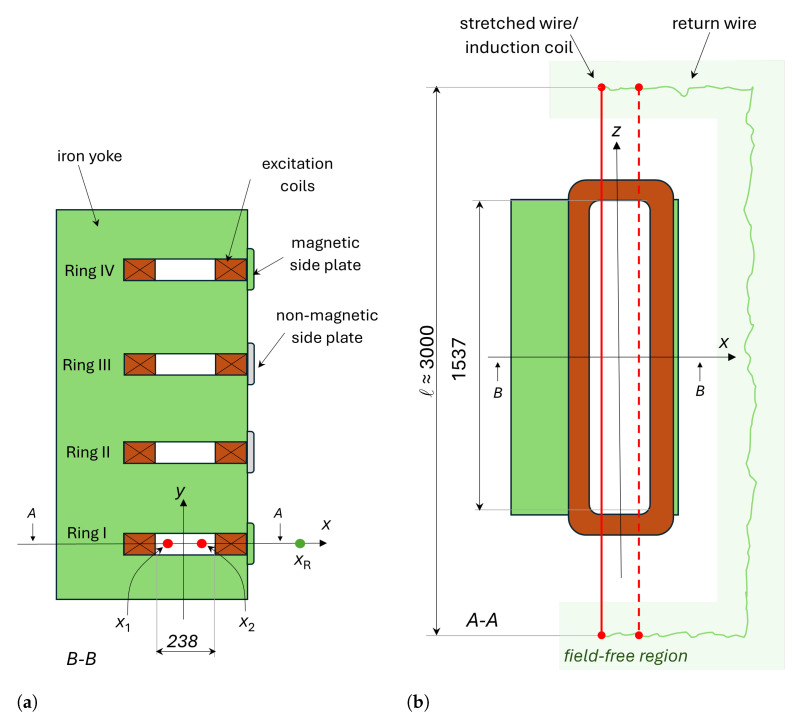
Schematic representation of the SSW system mounted in the four-gap PSB bending dipole magnet, where each gap corresponds to a different accelerator ring and all lengths are given in millimeters. Red lines denote the stretched wire, while green shows the return wire closing the flux loop. The induction coil can be placed along the same path followed by the stretched wire, being only slightly shorter (although both its ends still reach into the field-free region). (**a**) Front view. (**b**) Top view.

The general operating principle of the static SSW method is well documented [10,11,12]. A conducting wire is stretched along the axis of the magnet and then displaced along a known trajectory while the magnet current *I* remains constant. The path of displacement of the wire depends on the magnet type and the harmonics of the field that are measured, ranging from a simple segment to a complex elliptical rotation [13]. For the present case study, we shall consider a straight dipole magnet, as shown in Figure 3. The wire is stretched between two symmetric linear stage supports over a length *ℓ* parallel to the longitudinal *z* axis. The wire lies in the magnet’s midplane y=0 and is translated only along the *x* axis by the linear stages. Since we are dealing with a relatively short magnet, we can ignore the impact of gravity-induced sag. The ends of the wire and the return wire must be sufficiently far from the magnet to lie in a field-free region, where stray and environmental fields can be ignored. As a result, the flux linked through the wire loop at any time can be expressed as follows:

(4) $$\Phi \left(X\left(t\right),X_{R},I\right)=\int_{-\frac{\ell }{2}}^{+\frac{\ell }{2}}dz\int_{X(t)}^{X_{R}}B_{y}\left(x,0,z,I\right)\;dx$$

where X(t) and $X_{R}$ are the transverse positions of the stretched and return wire, respectively. Under the assumptions made, the flux does not depend either on the precise length of the stretched wire, or on the exact path followed by the return wire [14].

A static SSW measurement consists of the wire moving from $X_{1}$ to $X_{2}$ at an approximately constant speed dX/dt. According to Faraday’s law, the induced loop emf $V_{s}$ depends only on position and velocity:

(5) $$V_{s}\left(t\right)=-\frac{\partial \Phi }{\partial t}=\;\frac{dX}{dt}\int_{-\frac{\ell }{2}}^{+\frac{\ell }{2}}B_{y}\left(X\left(t\right),0,z,I\right)\;dz$$

In a good field region of a highly uniform dipole, the integral term is essentially constant, and the voltage is simply proportional to the stretched wire velocity, which, therefore, should be as high as possible to improve the signal-to-noise ratio (SNR). By digitally acquiring Equation (5) and then integrating it numerically with respect to time, we can derive the expression of the flux $\Delta \Phi_{s}$ linked through the rectangular surface swept by the stretched wire:

(6) $$\Delta \Phi_{s}\left(X_{1},X_{2},I\right)=-\int_{t_{1}}^{t_{2}}V_{s}\left(u\right)\;du=\Phi \left(X_{2},X_{R},I\right)-\Phi \left(X_{1},X_{R},I\right)=\int_{-\frac{\ell }{2}}^{+\frac{\ell }{2}}dz\int_{X_{1}}^{X_{2}}B_{y}\left(x,0,z,I\right)\;dx$$

where *u* is the integration variable, $X_{1}=X\left(t_{1}\right)$, and $X_{2}=X\left(t_{2}\right)$. In principle, this result does not depend upon the exact law of motion X=X(t) followed by the wire. In practice, however, the wire will vibrate every time it is accelerated, and the integration interval must be adjusted to allow the vibrations to damp out. The need to curb the velocity and acceleration of the wire may represent a limitation of the method, especially in the case of fast-cycled magnets such as the PSB dipole, where the duration of current plateaus is severely constrained.

Finally, the average field can be obtained according to Equation (2) as

(7) $${\bar{B}}_{s}\left(I\right)=\frac{1}{l_{m}d}\lim_{d\to 0}\Delta \Phi_{s}\left(-\frac{d}{2},\frac{d}{2},I\right)$$

where $d=X_{2}-X_{1}$ represents the stroke of the wire’s movement, which, in this paper, we will always take to be symmetric with respect to x=0. However, the magnitude of the integrated flux and, hence, its accuracy tend to vanish as d→0. In practice, an optimal wire stroke $d^{*}$ can be defined as a compromise between measurement accuracy and the additional error due to field nonuniformity, which must be found experimentally as discussed in Section 4.1, to obtain

(8) $${\bar{B}}_{s}\left(I\right)=\frac{1}{l_{m}d^{*}}\Phi_{s}\left(-\frac{d^{*}}{2},\frac{d^{*}}{2},I\right)$$

### 2.2. Pulsed SSW

The pulsed SSW method subtracts two dynamic measurements at the positions of the wire $X_{1}$ and $X_{2}$. The voltage induced in the wire loop when the magnet excitation current is changing while the wire position is kept fixed is given by

(9) $$V_{p}\left(t\right)=-\frac{\partial \Phi }{\partial t}=\;\frac{dI}{dt}\int_{-\frac{\ell }{2}}^{+\frac{\ell }{2}}dz\int_{X}^{X_{R}}\frac{\partial }{\partial I}B_{y}\left(X,0,z,I\left(t\right)\right)\;dx$$

By acquiring and integrating Equation (9) we can obtain the change between the initial and final linked fluxes:

(10) $$\Delta \Phi_{p}\left(X,X_{R},I_{0},I\left(t\right)\right)=-\int_{t_{0}}^{t}V_{p}\left(u\right)\;du=\Phi \left(X,X_{R},I\left(t\right)\right)-\Phi \left(X,X_{R},I_{0}\right)$$

where $I_{0}=I\left(t_{0}\right)$. In principle, the flux change depends only upon the initial and final states, much like in the case of static wire. However, the nonlinear relationship between current and field often makes it necessary to implement suitable measures to improve the reproducibility of results. Most importantly, the change between minimum and maximum current should always be monotonic to avoid switching to a different branch of the magnetic hysteresis loop. For the same reason, the rate of change of the current should also be constant to ensure reproducible losses. As a result, it is strongly recommended that a fixed current cycle I(t) be repeatedly applied at $X_{1}$ and $X_{2}$. The results shall then be subtracted numerically to finally obtain

(11) $$\begin{array}{c} \Delta^{2}\Phi_{p}\left(X_{1},X_{2},I_{0},I\right)=\Delta \Phi_{p}\left(X_{2},X_{R},I_{0},I\right)-\Delta \Phi_{p}\left(X_{1},X_{R},I_{0},I\right)=\\ \int_{-\frac{\ell }{2}}^{+\frac{\ell }{2}}dz\int_{X_{1}}^{X_{2}}\left(B_{y}\left(X,0,z,I\left(t\right)\right)-B_{y}\left(X,0,z,I_{0}\right)\right)\;dx \end{array}$$

where the squared delta symbol represents the difference with respect to the position of a difference with respect to time. In other words, Equation (11) represents the change of the flux linked through a rectangular area equivalent to a virtual, single-turn fixed coil of width $d=X_{2}-X_{1}$. Similarly to Equation (8), the average field can be derived by moving the wire by the optimal stroke $d^{*}$, thus obtaining

(12) $$\Delta {\bar{B}}_{p}\left(I_{0},I\left(t\right)\right)=\frac{1}{l_{m}d^{*}}\Delta^{2}\Phi_{p}\left(-\frac{d^{*}}{2},\frac{d^{*}}{2},I_{0},I\left(t\right)\right)$$

where the Δ symbol emphasizes the fact that Equation (12) represents a difference between two excitation current levels. The accuracy of this result depends crucially on the reproducibility of the magnetic field, which, unfortunately, is not guaranteed, even when identical I(t) cycles are applied in succession. This is mainly due to the possible impact of the previous excitation history and, to a lesser degree, to common imperfections of the excitation current, such as high-frequency ripple or uncontrolled transients (glitches or overshoots). When a test campaign includes a variety of different magnetic cycles, as is the case in the present work, two well-known strategies can be applied to improve reproducibility: (1) systematic execution of one or more normalization precycles; and (2) forcing each current cycle to swing monotonically between fixed minimum and maximum values to remain always on the same hysteresis loop. Both techniques are used in the present work, as discussed in Section 3.

**Figure 4:**
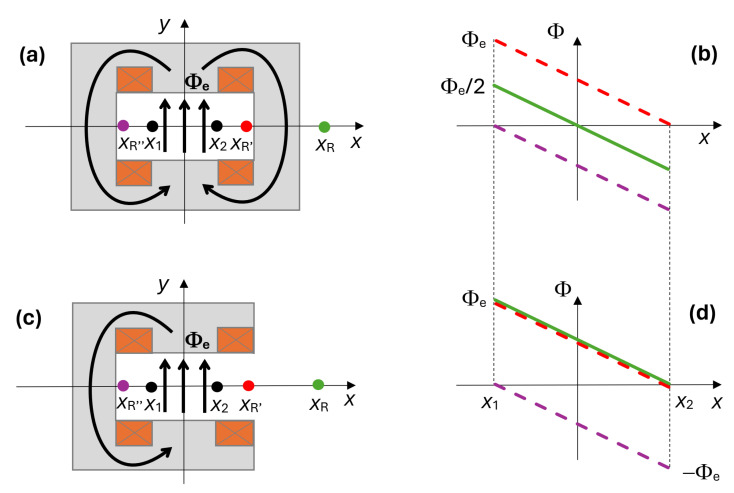
Impact of the position of the return wire, according to the type of magnet. (**a**): Magnetic flux in a symmetric window-frame dipole. (**b**): corresponding qualitative representation of the measured flux as the wire moves from $X_{1}$ to $X_{2}$. (**c**): Flux in an asymmetric C-shaped dipole. (**d**): corresponding measured flux. Green: external return wire ($X_{R}$). Purple: internal return wire ($X_{R^{\prime }}$).

Additional considerations must be made about the optimal position of the return wire, which may be placed externally or internally to the magnet. Let us consider two common cases, as shown in Figure 4: a window-frame-type dipole (top), where the return flux splits evenly between the two vertical legs of the iron yoke, and a C-shaped dipole (bottom), where one side is left open for easier access. For simplicity, we shall assume that all the magnetic flux generated by the excitation coils $\Phi_{e}$ is captured as the wire moves from $X_{1}$ to $X_{2}$. We find the following:

- **Window-frame dipole** (inset a, b): When the return wire is external to the magnet ($X_{R}$), the measured flux changes antisymmetrically with respect to the origin, reaching, at both ends, the same absolute value $|\Phi \left(X_{1},X_{R},I\right)|\;=\;|\Phi \left(X_{2},X_{R},I\right)|\approx \frac{1}{2}\Phi_{e}$. If, instead, the return wire is inside the magnet gap at either position $X_{R^{\prime }}$ or $X_{R^{\prime \prime }}$ due to the symmetry of the yoke, the linked flux will be ∼0 at one end and reach the maximum value ∼$|\Phi_{e}|$ at the other.
- **C-shaped dipole** (inset c, d): If the return wire is placed on the side where the yoke is open, either externally ($X_{R}$ ) or internally ($X_{R^{\prime }}$ ) to the gap, the measured flux will behave identically i.e., it will swing from the maximum value ∼$|\Phi_{e}|$ to ∼0; if, instead, the return is moved on the closed side of the yoke ($X_{R^{\prime \prime }}$), the sign of flux will be opposite as the curve Φ(X) is shifted by $-\Phi_{e}$.

For a static SSW measurement, these differences are irrelevant since the results depend only on the initial and final positions of the wire. Instead, in the case of pulsed SSW, it is important to maximize the peak value of the measured flux to gain dynamic range, which may improve the final accuracy, provided the acquisition chain can be adapted accordingly. For a C-shaped dipole, the maximum dynamic range is always guaranteed, while for a window-frame type, either internal position leads to a factor ∼2 improvement. The case of the PSB dipole corresponds to an intermediate situation closer to the C-shaped design since the flux returns on the x>0 side through a relatively thin plate. For the present case study, the return wire was fixed externally, and the resulting flux curves, which retain the same sign throughout the wire movement, are shown in Figure 4. Running the return wire internally may provide an additional advantage linked to lower EM interference pick-up due to the smaller loop area (see also the discussion in Section 4.3). Should one choose this configuration, fixing the mechanically stable wire is paramount since even very small movements in the high-field region may perturb the voltage readout.

### 2.3. Combined SSW

Embodiment in the same physical setup gives the unique opportunity to combine static and pulsed SSW measurement procedures to derive the absolute field, even under dynamic excitation conditions (c-SSW method). Assuming that DC excitation at $I_{0}$ is possible, we can simply add Equations (6) and (11) to define the combined flux as follows:

(13) $$\begin{array}{cc} \Phi_{c}\left(X_{1},X_{2},I\left(t\right)\right) & =\Delta \Phi_{s}\left(X_{1},X_{2},I_{0}\right)+\Delta^{2}\Phi_{p}\left(X_{1},X_{2},I_{0},I\left(t\right)\right)\\ \; & =\int_{-\frac{\ell }{2}}^{+\frac{\ell }{2}}dz\int_{X_{1}}^{X_{2}}B_{y}\left(x,0,z,I\left(t\right)\right)\;dx \end{array}$$

The average field can then be expressed as

(14) $${\bar{B}}_{c}\left(I\left(t\right)\right)={\bar{B}}_{s}\left(I_{0}\right)+\Delta {\bar{B}}_{p}\left(I_{0},I\left(t\right)\right)=\frac{1}{l_{m}d^{*}}\Phi_{c}\left(-\frac{d^{*}}{2},\frac{d^{*}}{2},I\left(t\right)\right)$$

As it incorporates the residual field, Equation (14) captures, in principle, the current magnetic state irrespective of the previous excitation history. However, as discussed in the previous section, the reproducibility of the two consecutive cycles necessary for the pulsed SSW is generally guaranteed only if the magnet is already on a stable hysteresis loop. A suitable number of normalization precycles, to be established experimentally for each magnet type, should be run before any measurement to ensure that is the case.

### 2.4. Induction Coil

Ideally, an induction coil can be represented by $N_{T}$ infinitely thin identical rectangular turns of length $l_{c}$ and width $w_{c}$. In practice, the width of each winding is affected by the pile-up of manufacturing tolerances, which increase with the total length and the number of turns. As a result, the average width of the windings should be considered instead of an unknown function $w_{c}\left(z\right)$ of the longitudinal position [15]. In general, the flux linked by the coil can be expressed as

(15) $$\begin{array}{c} \Phi_{\mathrm{coil}}\left(I\right)=N_{T}\int_{-\frac{l_{c}}{2}}^{+\frac{l_{c}}{2}}dz\int_{-\frac{w_{c}\left(z\right)}{2}}^{+\frac{w_{c}\left(z\right)}{2}}B_{y}\left(x,0,z,I\right)dx \end{array}$$

In the following, we shall assume that the length of the coil is sufficient to reach the field-free region at both ends, allowing us to use the same longitudinal integration limits as in the case of the stretched wire. In addition, we shall ignore the nonuniformity of the field across the coil, which is usually only a few millimeters wide. Under these assumptions, we can express the total flux linked through the coil as

(16) $$\Phi_{\mathrm{coil}}\left(I\right)=N_{T}\int_{-\frac{\ell }{2}}^{+\frac{\ell }{2}}w_{c}\left(z\right)B_{y}\left(0,0,z,I\right)\;dz$$

The flux at any given current level can be measured by integrating the voltage $V_{c}$ induced according to Faraday’s law in different ways. The following two methods were used in this work:

- **Flip-coil method**: The coil is first positioned flat in its central rest position inside the magnet’s gap (roll angle θ=0), then it is flipped upside down (θ=π) while measuring the induced voltage $V_{c}\left(t\right)$. The final flux linkage will be equal and opposite to the initial one, and we can derive the following:
  (17) $$\Phi_{\mathrm{coil}}\left(I\right)=\frac{1}{2}\int_{t(\pi )}^{t(0)}V_{c}\left(u\right)\;du$$
  where the integration bounds correspond to mechanically stable coil configurations. The result, in principle, does not depend upon the precise law of motion θ(t) followed, even if some translation is unwittingly superposed to the rotation. The main practical difficulty is turning the coil quickly enough to avoid the build-up of integrator drift while at the same time ensuring that the initial and final configurations are not offset in any direction. Mainly for this reason, a suitable nonmagnetic, nonconducting, rotating, mechanical support should be preferred to manual operation.

- **Fixed coil method**: We keep the coil fixed and instead ramp the current starting from zero up to its desired value, to obtain the flux change:
  (18) $$\Delta \Phi_{\mathrm{coil}}\left(I_{0},I\right)=\int_{t(I_{0})}^{t(I)}V_{c}\left(u\right)\;du$$
  This method is the most rapid and practical since it does not involve any coil handling; however, the measurement is blind to the initial flux $\Phi_{0}=\Phi \left(0\right)$. This is typically associated with a remanent field in the iron poles, which depends upon the previous magnetization history and can be of the order of a few milliteslas. Ideally, a demagnetization cycle should be applied before the measurement, but doing so requires bipolar power converters, which are not always available. In the alternative, $\Phi_{0}$ must be measured independently, for example, by the flip-coil method. Another independent measurement of $\Phi_{0}$ could be obtained with the static SSW method by scaling the results with the appropriate number of induction coil turns. However, the measured flux will not be entirely consistent with Equation (16), since the wire is much more straight than the coil windings, at least horizontally. Fortunately, the resulting error is often negligible since the integrated remanent field is very small, typically at least two orders of magnitude below the lowest level of interest (corresponding to beam injection). The value thus obtained is then added to the pulsed measurement to derive the absolute flux:
  (19) $$\Phi_{\mathrm{coil}}\left(I\right)=\Phi_{0}+\Delta \Phi_{\mathrm{coil}}\left(0,I\right)$$
  The same $\Phi_{0}$ can be reused multiple times for fixed coil measurements at different current levels. Doing so, however, requires that a stable hysteresis cycle be followed to ensure repeatability, as we have performed in the present work.

Regardless of the method followed, a straightforward and accurate derivation of the average field, such as in Equation (8) or Equation (12), is not possible due to the coil width being affected by a large uncertainty. We shall therefore define a suitable calibration parameter and the effective coil width as follows [16]:

(20) $$\begin{array}{c} w_{\mathrm{eff}}\left(I\right)=\frac{\Phi_{\mathrm{coil}}\left(I\right)}{l_{m}\bar{B}\left(I\right)}=\frac{N_{T}\int_{-\frac{\ell }{2}}^{+\frac{\ell }{2}}w_{c}\left(z\right)B_{y}\left(0,0,z,I\right)\;dz}{\int_{-\frac{\ell }{2}}^{+\frac{\ell }{2}}B_{y}\left(0,0,z,I\right)\;dz} \end{array}$$

which can be interpreted as the average coil turn width, weighed with the local magnetic field, additionally incorporating the number of turns to simplify practical usage as a single calibration coefficient. The calibration of the coil depends, at the same time, upon its geometry and the longitudinal field profile of the magnet being measured. In iron-dominated magnets, the most common choice for particle accelerators is the shape of the longitudinal field profile, which is mainly a function of the excitation current. At high fields, saturation increases the flux leaking out of the iron yoke, leading to a relative increase in the field strength of the fringe field region and a subsequent flattening of the profile. Calibration of the effective width from Equation (20) requires an independent measurement of $\bar{B}$ in the same magnet with a reference method, such as any form of SSW. For example, omitting for simplicity the incorporation of the residual field in both coil and SSW measurements, the pulsed SSW method can be used to derive the effective width as

(21) $$\begin{array}{c} w_{\mathrm{eff}}\left(I\right)=\frac{\Delta \Phi_{\mathrm{coil}}\left(0,I\right)}{l_{m}\Delta {\bar{B}}_{p}\left(0,I\right)}=d^{*}\frac{\Delta \Phi_{\mathrm{coil}}\left(0,I\right)}{\Delta^{2}\Phi_{p}\left(-\frac{d^{*}}{2},\frac{d^{*}}{2},0,I\right)} \end{array}$$

We note that any nominally identical magnets belonging to a series production run can be used as a reference, with their relative differences usually being very small. Once the calibration has been obtained, the average field can finally be derived from Equation (20) as follows:

(22) $${\bar{B}}_{\mathrm{coil}}\left(I\right)=\frac{\Phi_{\mathrm{coil}}\left(I\right)}{l_{m}w_{\mathrm{eff}}\left(I\right)}$$

## 3. Test Setup

**Figure 5:**
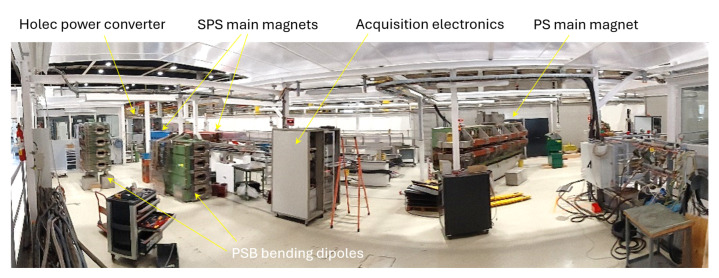
CERN test hall 867-R-29, showing two PSB bending dipoles being prepared for magnetic measurements. Thanks to the availability of the Holec power converter, this hall is a unique asset dedicated to testing potentially activated, high-pulsed-current magnets such as the other PS and SPS units shown.

**Figure 6:**
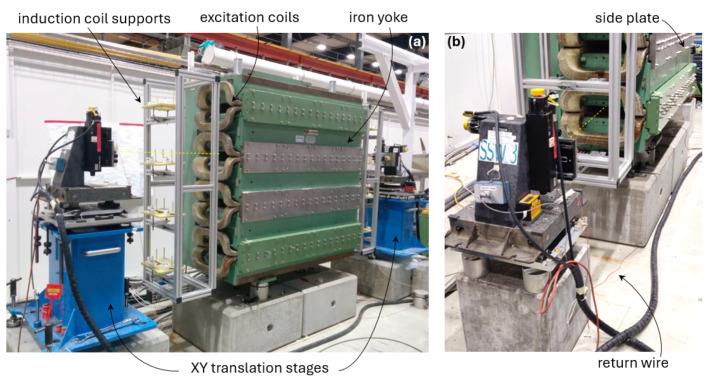
Test setup including the four-aperture PSB magnet and the SSW stages. (**a**) Illustration of the wire (dotted yellow line) stretched through the magnet between the two XY translation stages. (**b**) Detail of one stage placed in front of the lowest of the magnet’s apertures, i.e. the one used for the present work.

**Figure 7:**
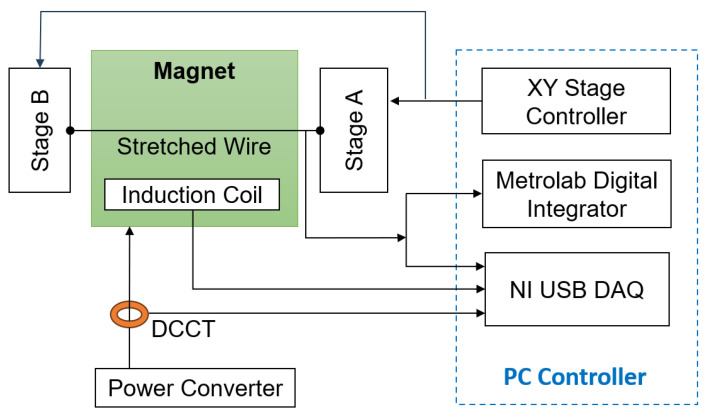
Schematic block diagram of the test setup.

The measurements discussed in Section 4 were obtained with the setup based on the PSB dipole magnet shown in Figure 5 and Figure 6 and represented schematically in Figure 7. The main design parameters and field quality requirements are listed in Table 1. The design of the PSB magnet is unique in that it consists of four vertically stacked apertures powered in series and sharing the same iron yoke, allowing for a four-fold increase in the number of protons that can be accelerated in parallel. In the context of this work, all apertures are functionally equivalent to a standalone magnet, and the measurements were performed in the bottom aperture.

### 3.1. SSW Setup

The setup used for this work is based on the systems originally developed at Fermilab to measure series LHC magnets [11,12]. The PC-controlled system consists of a 3 m long, ⌀125 μm beryllium copper wire stretched through the aperture and supported on either side by a Newport ESP 7000 XY translation stage, having a maximum ±75 mm stroke on both axes and mounted on a granite support. An additional motor keeps the wire under constant tension 8 N to limit the gravity-induced sagitta to about 0.1 mm [13]. The acquisition chain includes a Metrolab PDI-5035 voltage integrator [18] to measure the flux change induced by the displacement of the wire in static mode, as well as a National Instruments USB-6366 16-bit DAQ module to acquire the voltage induced in pulsed mode in both the wire loop and the coil up to 1MS/s, as well as the DCCT output measuring the excitation current. We point out that the large inductance of accelerator magnets usually limits the effective bandwidth well below 1 kHz; however, oversampling at high frequency is often very useful for estimating and then subtracting the low-frequency 1f noise components leading to integration drift.

### 3.2. Induction Coil

The integral induction coil used for this work is part of a batch of nominally identical units developed for the PSB dipoles. It consists of $N_{T}=70$ turns of copper wire wound around a $l_{c}=$ 2.75 m long, $w_{c}=$ 10 mm wide fiberglass core. For series measurements, these coils are installed inside grooves milled in G10 supports, held in place by an extruded aluminum profile structure, as shown in Figure 6. The coil used for this work was calibrated at I=1000 A by combining in (20) the flip-coil method (17) with a static SSW measurement, resulting in a calibration coefficient $w_{1000}=0.7083\;m$.

### 3.3. Magnet Powering

The PSB dipole was powered with a custom $6500\;A_{\mathrm{peak}}/3200\;A_{\mathrm{RMS}}$, ±200 V converter developed in 1997 by Holec Projects BV, NL, especially for testing CERN pulsed magnets [19]. This is a two-quadrant, current-controlled converter based on an 18 kV transformer and two parallel 6-pulse bridge thyristor rectifiers, preloaded with an additional 12-pulse thyristor rectifier at 150 A so as to be able to output stable controlled current down to zero. The combination of a passive LC filter having an 80 Hz cut-off with an active filter injection choke and amplifier enables suppression of the inherent 300 Hz ripple, while ensuring a good match to rapid transients in the input reference, up to a bandwidth of 10kHz. In particular, the active filter is designed to always approach the steady-state step response from below, i.e., without any overshoot that may switch the magnet’s response onto a different branch of the hysteresis loop. As a result, the overall accuracy of the output current is better than 23ppm of the full scale for loads up to 200mH and 120mΩ.

**Figure 8:**
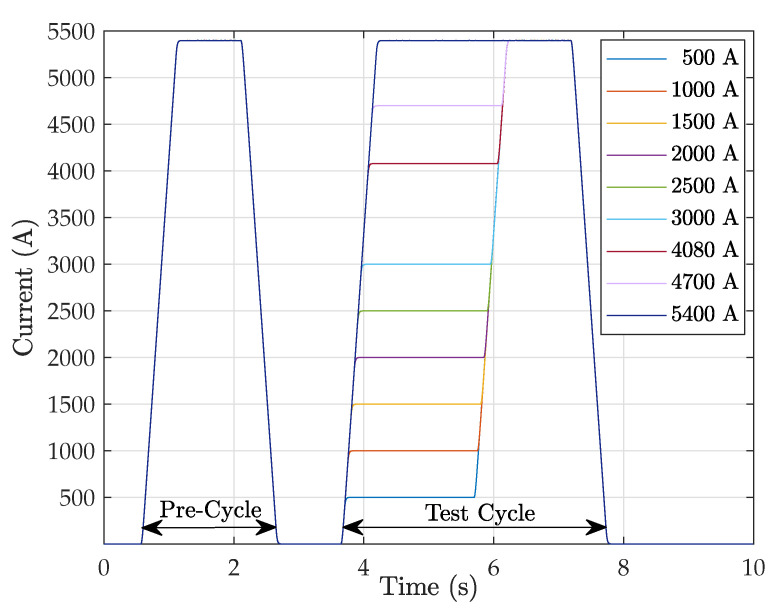
Excitation current waveforms used to power the PSB magnet.

The excitation current waveforms are plotted in Figure 8. The current was measured with a 10V/6000A TOPACC 1.0 Zero-Flux DCCT, also developed by Holec and implemented as an integral part of the current control loop of the converter [19]. The DCCT working principle is based on a high-turn-ratio secondary winding, powered by a feedback-controlled amplifier in order to cancel out the flux generated by the primary, represented by the current to be measured. In this way, the secondary current is used to derive the primary with combined DC offset and linearity errors below 5ppm with respect to the full scale, in addition to a long-term stability of 5ppm/year and a thermal stability of $0.25\;\mathrm{ppm}{/}^{\circ }C$. The dynamic performance of the DCCT can be expressed in terms of the maximum output slew rate, i.e., 1.5 V/μs, which corresponds to 900 A/μs. This is four orders of magnitude higher than the fixed ramp rate of the excitation cycles, that is, 10 kA/s, resulting in a completely negligible delay. The rated noise level of the DCCT from DC up to 10kHz is as low as 1.5ppm of full scale, i.e., about 10 mA. The level of random noise measured in the acquired signal is about 100 mA, i.e., about $3\;\cdot \;10^{-5}$ of the nominal peak value $I_{\max}=5400\;A$. This can be largely attributed to the cabling and acquisition system, and it remains well below the required accuracy.

The waveforms consist of a sequence of two cycles that start at I=0, reach $I_{\max}$, and then return to I=0. The first is a normalization precycle that has the function of improving the reproducibility of the magnet’s response, with a flat-top duration of 1 s. As a rule, normalization precycles should reach at least as high as the highest level of the cycles being run, due to the well-known wiping out property [20]. Typically, the number of normalization cycles necessary to ensure repeatability of the flat-top field within a given tolerance tends to decrease with the level of saturation, which asymptotically represents a uniquely defined reference state. However, based on our own experience, no general quantitative prescription can be formulated; instead, the number of repetitions should be established experimentally. In this case study, a single precycle was enough, plausibly due to the high level of saturation reached by the PSB magnet in operation.

After the precycles, the proper test cycle begins, introducing an intermediate plateau at a level ranging from 500 A to 5400 A. For measurements with pulsed SSW and fixed coil methods, the duration of the plateau was 2 s, which is enough to allow the eddy currents to decay (as discussed in Section 4.4). For measurements using the static SSW method, the duration of the plateau was extended to 120 s. This long duration is needed for at least three back-and-forth wire sweeps, which are averaged to improve the SNR. However, extended DC powering of the PSB dipole is possible only up to a maximum of 3000 A to keep the temperature of the excitation coils below a safe level, typically 60 °C. In all cases, the absolute value of the ramp rate was fixed to ensure stable power converter operation.The measured stability and reproducibility of the current plateaus is about ∼$10^{-5}$. The strategy of combining a precycle with a fixed maximum current level proved very effective in controlling the effects of hysteresis, and the residual field measured with the static SSW method, $l_{m}{\bar{B}}_{0}\;=\;0.54\;\mathrm{mTm}$ (i.e., $3\;\cdot \;10^{-4}$ with respect to the nominal field), was found to be reproducible throughout the test campaign.

## 4. Measurement Results and Analysis

In this section, we analyze and compare the results obtained with the three measurement methods. A detailed summary of the main results is given in Table 5.

### 4.1. Field Uniformity

**Figure 9:**
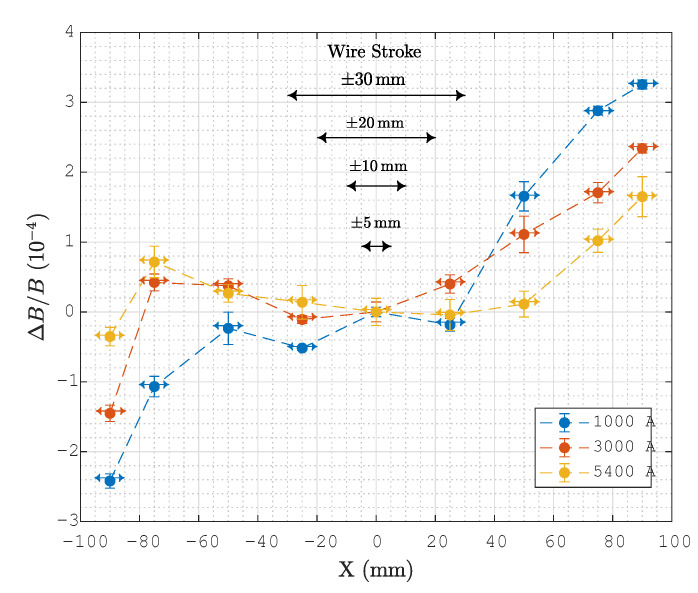
Field uniformity relative to the value at 0 mm, as measured using the fixed coil method. Vertical error bars represent the standard deviation over three measurements, while the horizontal arrows represent the induction coil’s width of 10 mm. Dashed lines denote positions of $X_{1}$ and $X_{2}$ for the different SSW configurations under test.

The uniformity of the transverse field was measured with the fixed coil method as a preliminary step in order to determine the optimal range of the SSW stroke. Measurements were taken at x=0,±25,±50,±75, and ±90 mm for I=1000,3000, and 5000 A, and the results normalized to the central values, expressed in terms of

(23) $$\frac{\Delta B}{B}\left(x,I\right)=\frac{\int_{-\infty }^{\infty }B_{y}\left(x,0,z,I\right)dz}{\int_{-\infty }^{\infty }B_{y}\left(0,0,z,I\right)dz}-1,$$

are plotted in Figure 9 and listed in Table 2. The table also provides the lowest-order normal field harmonics $b_{n}$ [5], obtained by performing a least-squares fit of the measured field profiles over the interval ±90 mm. The uniformity at all current levels is equal to or better than $3.4\;\cdot \;10^{-4}$, which is within the tolerance given in Table 1. The field error is dominated by the sextupole and decapole components, which are allowed by the nominal symmetry of the main dipole component. However, because of the actual asymmetry of the construction, smaller quadrupole and octupole components are also present, as can be observed in the vicinity of the axis. These and all other even-order harmonic field components do not affect the measurement of the dipole, as long as the SSW stroke remains symmetric with respect to the origin.

In order to obtain a high level of integrated signal, it is always preferable to sweep the stretched wire across the widest possible range, provided the nonuniformity of the field does not impact the result. In our case, we find that the stroke $d^{*}=60\;\mathrm{mm}$, corresponding to the extreme positions of the wire ±30 mm, gives a maximum relative error with respect to the central value equal to $0.2\;\cdot \;10^{-4}$, which represents a reasonable compromise. In the following sections, a detailed comparison of the results obtained with shorter strokes of ±10,±20, and ±5 mm is given.

### 4.2. Pulsed SSW Results

**Figure 10:**
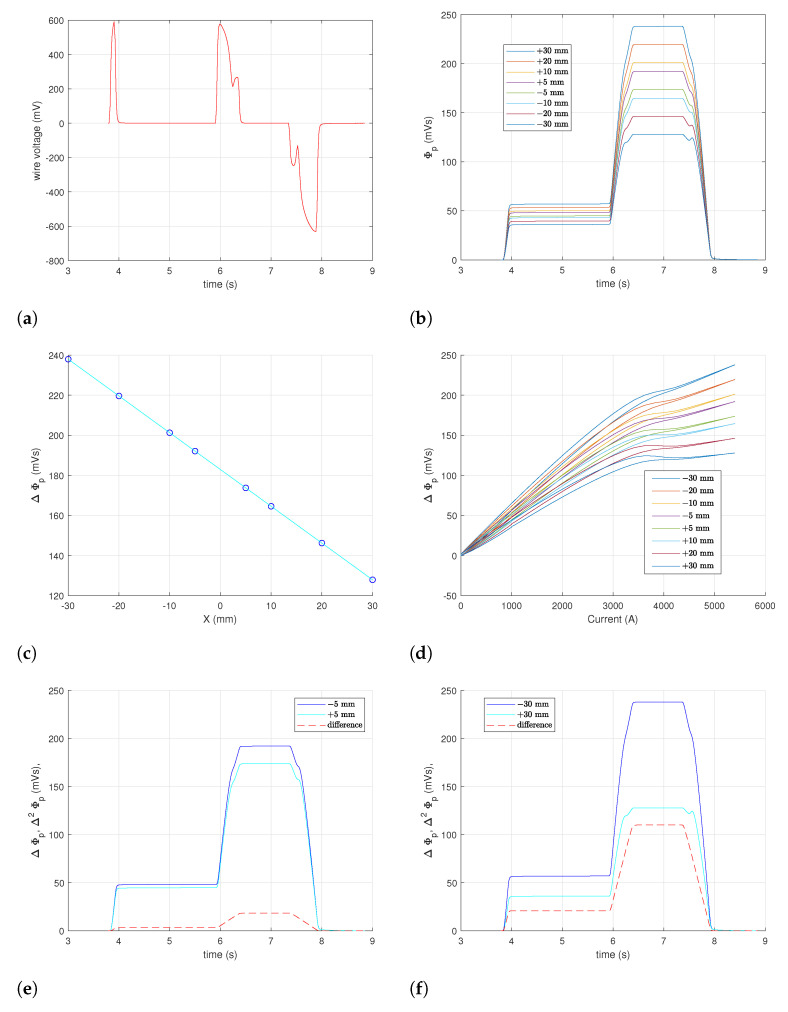
Examples of pulsed SSW measurements of a 1000 A plateau test cycle, taken at eight wire positions between X=±30 mm. (**a**) Example of raw output voltage at X=+5mm; (**b**) Integrated flux $\Delta \Phi_{p}$. (**c**) Peak flux at t=7.2 s vs. wire position. (**d**) Magnetization curves of $\Delta \Phi_{p}$ vs. current. (**e**) Difference of $\Delta \Phi_{p}$ at ±5mm. (**f**) Difference of $\Delta \Phi_{p}$ at ±30mm.

Let us analyze in detail an example of the flux measured with the pulsed SSW method. As illustrated in Figure 10, we focus on a test cycle with a 1000 A plateau and dynamic measurements taken plotted in the positions X=±5,±10,±20, and ±30 mm. In particular, measurements at $X_{1}=+5$ mm, $X_{2}=-5$ mm correspond to the nominal width of the induction coil and can be used to make a direct comparison, as discussed in Section 4.6.

Inset (a) shows an example of the voltage output of the wire loop. Thanks to the high field ramp rate, even a single-turn loop provides a peak voltage of the order of one volt, which, combined with the 16-bit acquisition, is enough to guarantee accurate and drift-free integration of the flux. The flux $\Delta \Phi_{p}$ integrated at all wire positions is plotted vs. time in the inset (b). Due to the high uniformity of the field in the interval scanned by the wire, the curves scale with a good approximation linearly with respect to the wire position. The flux at the end of the cycle flat-top, plotted in inset (c), varies linearly from about 240 to 130 mVs, with a relative residual RMS of the order of $10^{-5}$. This should be compared with the ideal expectation illustrated in Figure 4d, considering that the measurement range ±30 mm captures only a fraction of the total flux in the 180 mm gap width.

The curves $\Delta \Phi_{p}\left(t\right)$ exhibit a peculiar kink at high field, both at the end of the ramp-up and at the beginning of the ramp-down. By instead plotting the flux as a function of the excitation current, as shown in inset (d), it appears that the kink is due to the saturation of the yoke. When the wire is in the rightmost position, X=+30 mm, the fall-off due to saturation above ∼3500 A is about 40% with respect to the approximately linear behavior at low current. This level of saturation is much higher than what is observed in the center of the gap (see Section 4.6) because, in this case, the flux measured by the wire loop is dominated by the contribution of the thin side plates. Finally, according to (11), the measured flux curves were subtracted numerically pairwise to obtain the virtual-coil equivalent $\Delta^{2}\Phi_{p}$. Two examples of the results for the cases X=±5 and ±30 mm are plotted in the insets (e) and (f), respectively. The flux difference curves resemble the current waveform much more closely than the $\Delta \Phi_{p}$ curves since the kinks cancel out, as expected. The peak values of $\Delta^{2}\Phi_{p}$ are reported in Table 5 for the case X=±30 mm.

### 4.3. Noise Analysis

The noise of the fixed coil and pulsed SSW was analyzed in the frequency domain as a function of the stroke width *d* and acquisition sampling rate for a magnetic cycle with a 5400 A plateau. The results, summarized in Table 3, are expressed in terms of the fraction η of the power spectrum that can be attributed to extraneous noise, defined as

(24) $$\eta =\sqrt{\frac{\int_{f_{m}}^{\infty }{\left|\Psi \left(f\right)\right|}^{2}df}{\int_{0}^{\infty }{\left|\Psi \left(f\right)\right|}^{2}df}}$$

where $\Psi \left(f\right)=F(\bar{B}\left(t\right))$ is the Fourier transform of the measured field, its modulus squared provides the power spectrum, and $f_{m}\approx 80\;\mathrm{Hz}$ is the highest appreciable frequency content of the magnetic cycle waveforms. In practice, (24) is evaluated via the FFT of the discrete field signal, and integration is truncated at the Nyquist frequency.

**Figure 11:**
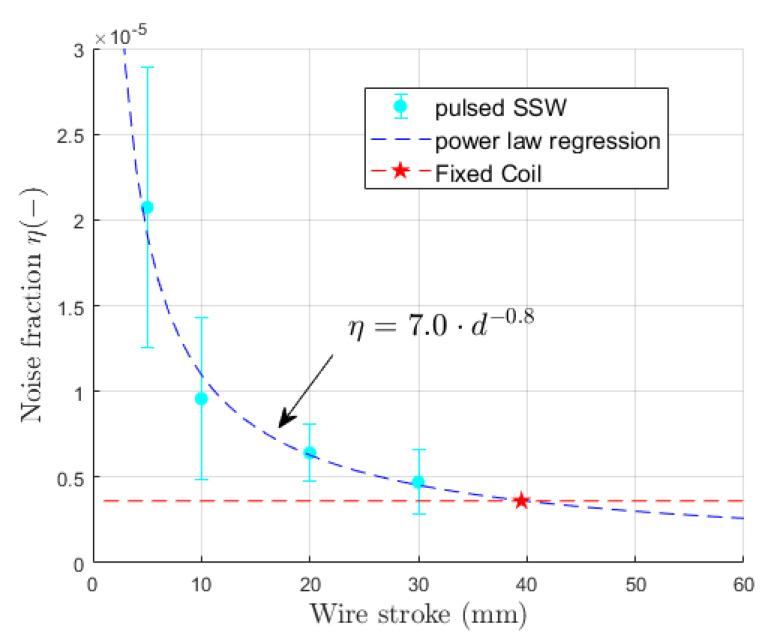
Effect of displacement width on the p-SSW setup’s noise content for a 5400 A plateau cycle. Vertical error bars represent the standard deviation over 3 repetitions.

Overall, the noise level is very low, being on the order of a few $10^{-5}$ for the fixed coil and the pulsed SSW. The results obtained at the sampling rate of 1MS/s are plotted as a function of the wire stroke in Figure 11. The pulsed SSW data points fit roughly a relationship of inverse proportionality with respect to *d*, as one might expect, assuming that the noise level in the raw acquisition remains constant, while the measured flux $\Delta \Phi_{p}$ is proportional to *d*. The noise content of the fixed coil measurement is $\eta =3.6\;\cdot \;10^{-6}$, which is equivalent to an ideal wire stroke as wide as ∼39 mm. The pulsed SSW results are clearly penalized by the intrinsic limitations of the method, which are (a) lower signal levels due to the single-turn nature of the wire loop, and (b) the difference between two separate measurements adds noise components (including quantization noise and excitation current ripple) and common modes quadratically, instead of canceling them out.

**Figure 12:**
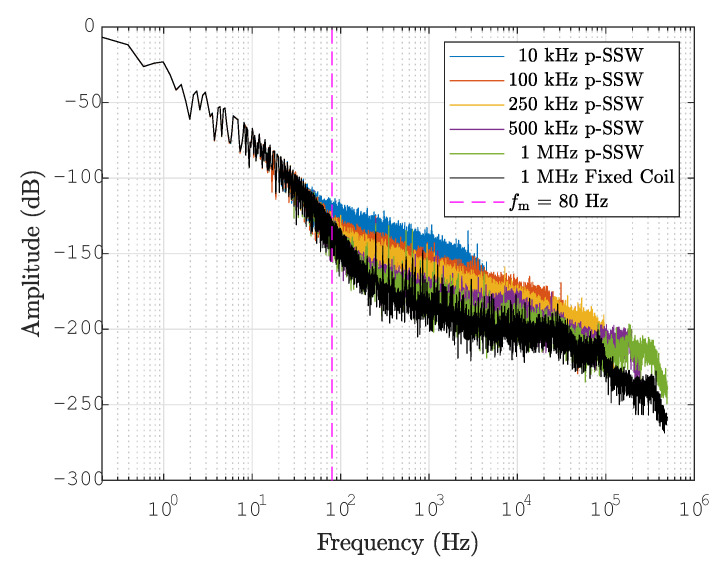
Amplitude spectrum of p-SSW and fixed coil, acquired at different sampling rates. The part of the spectrum above $f_{m}$ is attributed to noise only.

Further insight into the relative performance of the two methods can be gained from the amplitude spectra plotted in Figure 12, which include the fixed coil acquired at 1MS/s and the ±30mm pulsed SSW acquired at sampling rates down to 10kS/s. All measurements were carried out with the same anti-aliasing analog filter set at the maximum Nyquist frequency, that is, 500kHz. For comparison, pulsed SSW signals were also downsampled by the decimation of the 1MS/s acquisition in the post-processing phase. The spectrum of the induction coil (in black) is characterized by an initial steep slope, up to ∼200 Hz, associated with the shape of the magnetic cycle, followed by a roll-off slope about −20dB/decade, indicating the effect of the coil as a first-order RL low-pass filter. The slope approximately doubles above ∼80kHz, marking the lowest-order resonance associated with the self-capacitance of the coil. At the same sampling frequency of 1MS/s, the pulsed SSW spectrum (in green) exhibits a first-order slope similar to that of the coil but shifted about 15dB higher, consistently with the lower L/R of the wire, leading to a higher cutoff frequency. By lowering the sampling rate down to 10kHz while intentionally maintaining the same anti-aliasing filter, we observe that the noise of the pulsed SSW increases by as much as ∼50 dB. This result does not depend appreciably on the way downsampling is achieved, either by lowering the ADC settings or by decimation of the signal acquired at the highest rate in post-processing. This increase can be entirely ascribed to aliasing and demonstrates how the single-turn wire loop behaves as a very effective antenna picking up high-frequency disturbances, which, instead, are filtered out by the induction coil. This result highlights the importance of reducing the setup susceptibility to broadband noise, not only, of course, by including an appropriate anti-aliasing filter, but also by minimizing the surface area of the return loop.

### 4.4. Eddy Current Decay Transient

**Figure 13:**
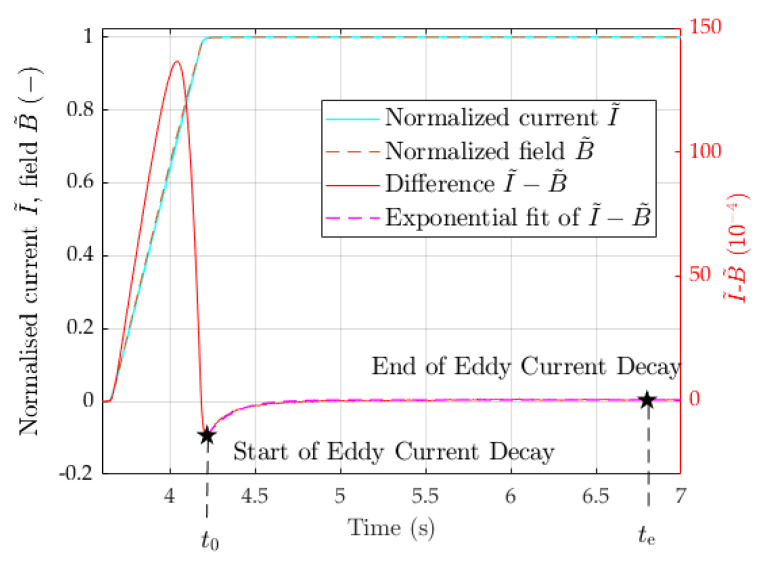
Normalized current and field showing the eddy current decay.

**Figure 14:**
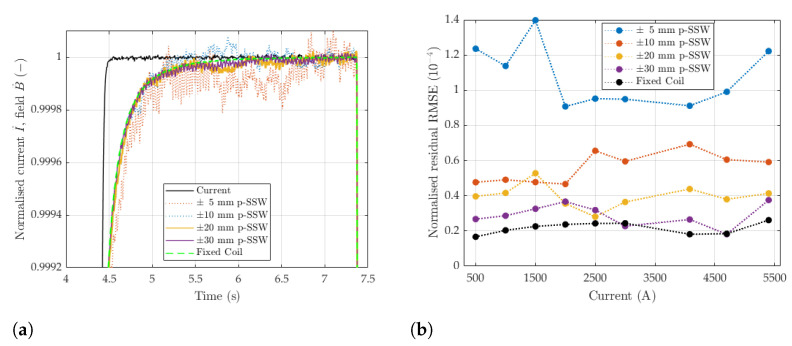
(**a**) Normalized magnetic field measured on the 5400A test cycle. (**b**) Normalized RMS residual calculated across all measurement plateaus. Values are relative to corresponding magnetic field.

The measurement of the amplitude and time constant of eddy current decay transients is necessary to characterize magnets for operation, as well as to establish a time interval on the cycle plateaus where the comparison between static and dynamic measurements is meaningful. The magnetic cycles used for our tests, as those generally used for synchrotron operation, are composed of successions of linear current ramps and plateaus where eddy currents develop at the start of ramps, and decay at their end. As an example, we focus on the transient at the end of a ramp-up to 5400 A, depicted in Figure 13. The normalized current $\tilde{I}$, normalized field $\tilde{B}$, and their difference δ are defined as follows:

(25) $$\tilde{I}\left(t\right)=\frac{I(t)}{I(t_{e})},\;\;\;\;\tilde{B}\left(t\right)=\frac{\bar{B}\left(t\right)}{\bar{B}\left(t_{e}\right)}\;,\;\;\delta \left(t\right)=\tilde{I}\left(t\right)-\tilde{B}\left(t\right)$$

where the average field is measured with the fixed coil, and the time $t_{e}$ represents steady-state conditions, when the eddy current can be assumed to have fully decayed. The difference δ represents the relative impact of nonlinear effects, and it can be clearly seen to undergo an exponential decay according to the following expression:

(26) $$\delta \left(t\right)=-A_{0}e^{-\frac{t-t_{0}}{\tau }}$$

where $t_{0}=4.2\;s$ is the end of the ramp-up, $A_{0}$ represents the peak relative field error, and τ is the time constant of the decay [14]. Equation (26) has been fitted with least squares to measurements taken with the induction coil and the pulsed SSW method with the wire stroke range ±5,±10,±20, and ±30mm, and the results are reported in Table 4. The variance of the fitted parameters is lowest for the induction coil and decreases as the SSW wire stroke increases, which is consistent with the levels of signal noise appearing in the example shown in Figure 14a. This result is also in agreement with the findings reported in Figure 11, which shows the signal noise decreasing with *d*.

Measurements were repeated for cycles with plateaus at different levels, and the results are also listed in Table 4. The fitted parameters are reported in Table 5, and the RMSE residuals of the exponential fitting are plotted in Figure 13 as a function of the integrated field. The accuracy of the fitting improves with increasing *d* and, even more substantially, with the inverse of the field level.

### 4.5. Comparison with Static SSW

The performance of the combined SSW method was compared with that of the static SSW, which is the reference method for DC conditions. For both methods, the integrated field measured with a stroke of ±30 mm is reported in Table 5 for each plateau level from 500 to 3000 A, along with the standard deviation over three consecutive repetitions. The relative difference over all current levels has a systematic (average) value of $3.8\;\cdot \;10^{-5}$, while the standard deviation is $1.2\;\cdot \;10^{-4}$. These results confirm that the performance of the combined SSW method meets operational requirements, which are typically of the order of $10^{-4}$.

**Figure 15:**
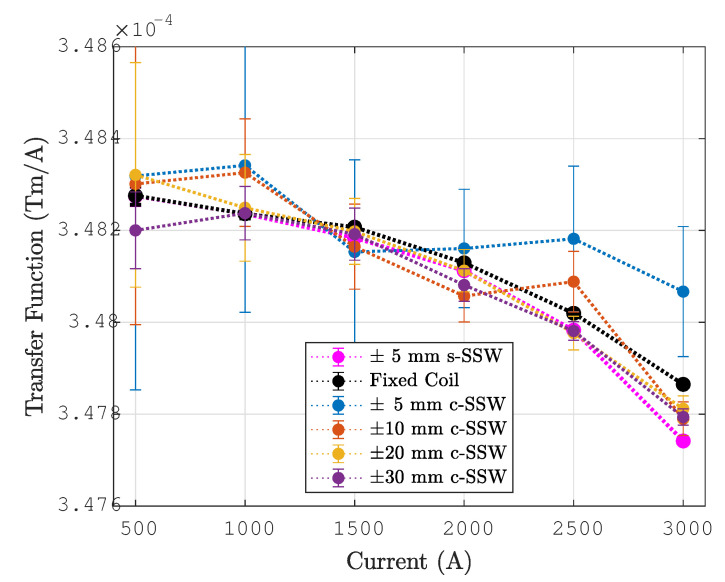
Transfer function measured with the fixed coil, static, and combined SSW in the current range allowing for DC powering. Error bars represent twice the standard deviation over three consecutive repetitions.

The comparison is also made in terms of the integral transfer function, illustrated in Figure 15 and defined as the ratio between the integral field and the current:

(27) $$TF\left(I\right)=l_{m}\frac{\bar{B}\left(I\right)}{I}$$

The transfer function is a very useful tool, not only because it allows machine operators to easily set the current necessary to achieve a given field, but also because it helps to visualize even minor deviations from the desired linear behavior, which ideally corresponds to a simple flat line. Moreover, by normalizing with respect to the current, the confounding impact of current reproducibility is eliminated inherently. This is reflected in the systematic relative difference with respect to the static SSW, which reduces to $2.1\;\cdot \;10^{-5}$, while the standard deviation is $1.3\;\cdot \;10^{-4}$.

**Table 5:** Summary of the main results obtained with the different measurement methods. All SSW measurements were performed with a ±30mm stroke.

Current (A)	Integral Field $l_{m}\bar{B}$ (Tm)		Integral Transfer Function $\frac{l_{m}\bar{B}}{I}\;\left(10^{-4}\frac{\mathrm{Tm}}{A}\right)$		Induction Coil Calibration $w_{\mathrm{eff}}$ (mm)	
	**s-SSW**	**c-SSW**	**s-SSW**	**c-SSW**	**s-SSW**	**c-SSW**
0	0.00054	-	-	-	-	-
500	0.17414±0.00001	0.1741±0.00005	3.4828±0.0002	3.4820±0.0008	-	708.27±0.24
1000	0.34824±0.00001	0.34823±0.00006	3.4824±0.0001	3.4824±0.0006	708.30±0.03	708.26±0.17
1500	0.52228±0.00001	0.52228±0.00008	3.4818±0.0000	3.4819±0.0006	-	708.27±0.17
2000	0.69623±0.00001	0.69616±0.00007	3.4811±0.0000	3.4808±0.0004	-	708.29±0.10
2500	0.86996±0.00001	0.86995±0.00005	3.4798±0.0000	3.4798±0.0002	-	708.29±0.06
3000	1.04323±0.00001	1.04338±0.00005	3.4774±0.0000	3.4779±0.0002	-	708.34±0.05
4080	-	1.41406±0.00006	-	3.4659±0.0001	-	708.43±0.03
4700	-	1.61743±0.00004	-	3.4413±0.0001	-	708.50±0.03
5400	-	1.83548±0.00006	-	3.3990±0.0001	-	708.56±0.03

### 4.6. Comparison with the Fixed Coil Setup

**Figure 16:**
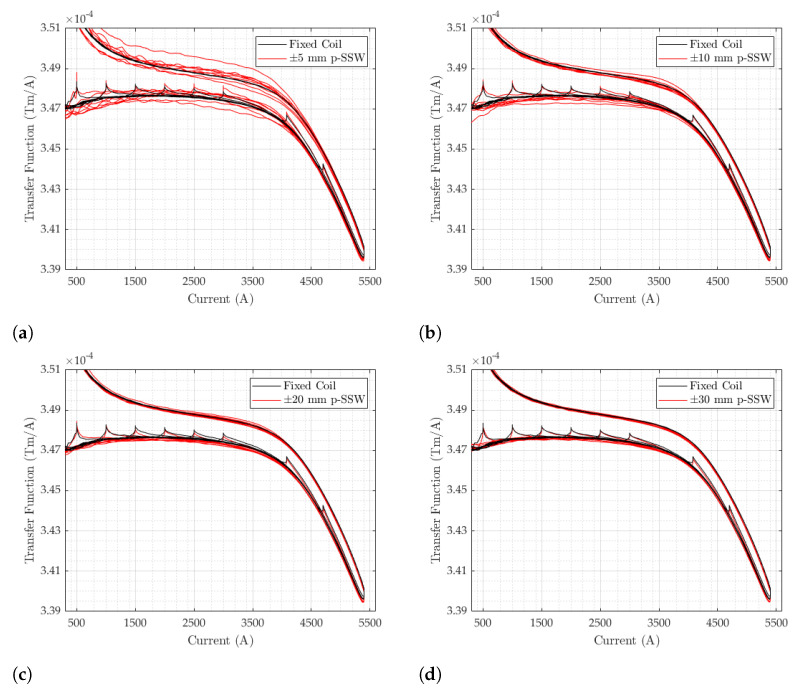
Transfer function of cycles with plateaus from 500 to 5400 A measured with the p-SSW and the fixed coil. (**a**) $w_{d}=\pm 5$ mm. (**b**) $w_{d}=\pm 10$ mm. (**c**) $w_{d}=\pm 20$ mm. (**d**) $w_{d}=\pm 30$ mm.

The comparison between pulsed SSW and fixed coil is analyzed in detail with the aim of deriving the coil calibration across the whole range of currents. We remark that adding or not the residual field to the pulsed SSW results has no impact, provided, of course, that the same is applied to the coil results. The transfer functions measured over the entire set of test cycles with the fixed coil and the pulsed SSW are plotted in Figure 16, separately, for the four-stroke lengths ±5,±10,±20, and ±30 mm. The calibration used for the fixed coil is that obtained with the flip-coil method.

As a general characteristic, the transfer function is approximately flat on the ramp-up between 1000 and 3500A, which is indicative of the desired linear response. At higher currents, the curve drops by about 2.3% due to iron saturation. This drop, measured in the magnet gap, depends on the average level of saturation of the whole iron yoke and is, therefore, one order of magnitude below the drop registered by individual $\Delta \Phi_{p}$ measurements, which capture the much higher level of saturation of the side plates. On the intermediate plateaus and on the flat-top of the excitation cycles, we observe an increase in the field at constant current due to the decay of the eddy currents that screen the field in the gap. As discussed in Section 4.4, steady-state conditions apply at the end of the decay transient and the corresponding values are listed in Table 5. The ramp-down of the hysteresis loop is not relevant for synchrotrons such as the PSB, as no beam is circulating. Both branches of the loop diverge as the current approaches zero, due to the presence of a residual field. All these features are captured equally well by both measurement methods. Consistent with the results reported in the previous sections, the reproducibility of the pulsed SSW improves dramatically as the stroke lengthens and the field increases. At injection (I≈1100 A), the relative standard deviation changes from 0.6% at ±5mm (Figure 16a) to 0.1% at ±30mm (Figure 16d), further dropping to 0.03% at 5400 A. This result confirms the choice of the wire stroke $d^{*}=60$ mm as the optimal one, which we also used as the basis to derive the coil calibration. The comparison between the two methods was carried out using the ratio ρ between the respective transfer functions, which can be expressed as follows:

(28) $$\rho \left(I\right)=\frac{TF_{\mathrm{coil}}\left(I\right)}{TF_{p}\left(I\right)}=\frac{{\bar{B}}_{\mathrm{coil}}\left(I\right)}{{\bar{B}}_{p}\left(I\right)}=\frac{d^{*}}{w_{1000}}\frac{\Delta \Phi_{\mathrm{coil}}\left(0,I\right)}{\Delta^{2}\Phi_{p}\left(-\frac{d^{*}}{2},\frac{d^{*}}{2},0,I\right)}$$

**Figure 17:**
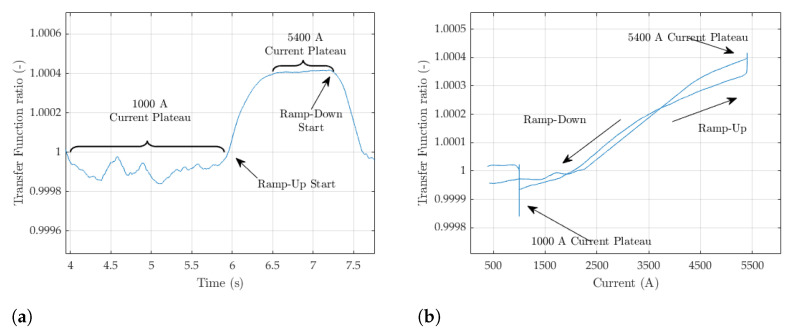
Ratio between the transfer functions measured with fixed coil and the ±30 mm pulsed SSW over a 1000 A test cycle. (**a**) As a function of time. (**b**) As a function of current.

The ratio corresponding to the ±30 mm pulsed SSW is plotted as a function of time in Figure 17a and of current in Figure 17b, where only the 1000 A plateau cycle is shown. As expected, at low current, ρ≈1, except for a discontinuity corresponding to the decay of the eddy currents on the plateau. However, as the current increases above 2000 A, the ratio increases almost linearly by as much as 0.04% at 5400 A, which denotes a substantial change in coil calibration with field level. The effective coil width can be derived by combining (21) and (29) to obtain

(29) $$w_{\mathrm{eff}}\left(I\right)=\rho \left(I\right)w_{1000}$$

**Figure 18:**
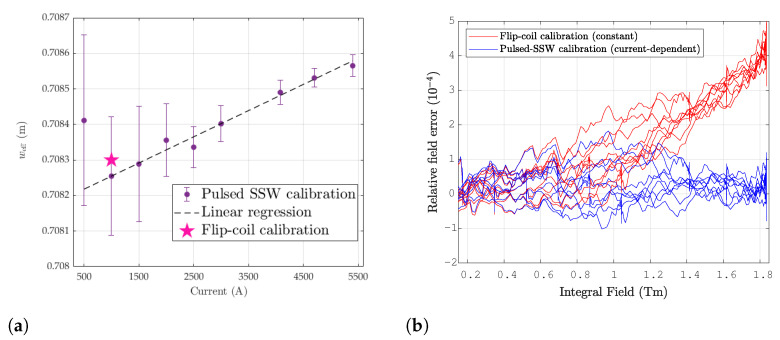
(**a**) Induction coil effective width derived from the difference with respect to ±30 mm combined SSW measurements. Vertical error bars represent 2-sigma standard deviation of over 9 measurements. (**b**) Relative difference between the test cycles measured with the fixed coil and ±30 mm c-SSW setups. The plot shows the improvement in accuracy due to the current-dependent $w_{\mathrm{eff}}\left(I\right)$ calibration.

The results are listed in Table 5 and plotted in Figure 18a, where the error bars represent the standard deviation over the set of nine test cycles. As a first approximation for practical use, the effective width can be reasonably well fitted by the following linear expression:

(30) $$w_{\mathrm{eff}}\left(I\right)=w_{0}+w^{\prime }\frac{I}{I_{\max}}$$

where $w_{0}=0.7082\;m$ and $w^{\prime }=4.0\;\cdot \;10^{-4}$. The initial value is very close to the one obtained by the flip-coil calibration method, i.e., 0.7083m. This could be expected to apply throughout the linear part of the range. However, an accurate evaluation in this region is impeded by the high standard deviations due to low signal levels. The increase in the effective width at a high field can be explained by one, or both, coil ends being marginally wider than the central part, thus being able to capture more of the high-field leakage due to saturation. Such a geometrical imperfection is actually often observed in coils wound with multiconductor cables due to their stiffness, which makes it relatively difficult to bend the, around the $90^{\circ }$ corners at the ends of the winding form. Regardlesss of the physical origin, using the calibration (30) allows the elimination of any systematic current-dependent discrepancy with respect to pulsed SSW measurements, as shown in Figure 18b.

## 5. Conclusions and Future Work

In this paper, we presented a novel method for measuring the dynamic integral field of an accelerator magnet using the SSW measurement bench. The pulsed SSW method was shown to accurately characterize the integral field of the CERN PSB bending dipole over its entire range of operation, which was not possible using the previous static implementation. Similar to static SSW, the measurement uncertainty was shown to depend strongly on the wire stroke. Therefore, we suggest identifying an optimal stroke width that meets the measurement precision without compromising the uncertainty from the field uniformity. The pulsed SSW shows no significant systematic difference compared to the static SSW, with a relative RMS difference of less than $2\;\cdot \;10^{-4}$ across their shared field range. This level of performance is closely comparable to that of other instrumentation and generally up to beamline requirements.

No implementation of the SSW method can clearly compete with a multiturn induction coil in terms of SNR, immunity to high-frequency external perturbations, and practicality of use in a context requiring multiple dynamic measurements of different magnetic cycles. However, the pulsed SSW method offers crucial advantages with its extreme geometrical adaptability and accuracy of the width over the flux-capturing area. Moreover, the dynamic performance was shown to improve with the rate of change of the magnetic field under conditions that make static SSW measurements more difficult. Overall, this method represents a valid general alternative to the static SSW, demonstrating its viability as a metrological reference when calibrating an induction coil as a function of the iron core’s saturation level. Future planned developments include widening the wire stroke into the nonuniform field region and the estimation of the field harmonic errors; upgrading the signal conditioning electronics to improve EMI robustness and SNR; and extending the method to quadrupole and higher-order multipole magnets. 

## Figures and Tables

**Table 1:** Main geometrical and electrical design parameters of the PSB bending dipole, along with the principal magnetic field quality requirements [17].

Parameter	Value	Unit
Iron core length	1537	mm
Gap height	70	mm
Gap width	238	mm
Good field region width	160	mm
Excitation turns/gap	12	-
Total excitation resistance	9.9	mΩ
Total excitation inductance	41.5	mH
Peak excitation current	5400	A
Integrated dipole tolerance $\frac{\bar{B}}{{\bar{B}}_{max}}-1$	$\pm 5\;\cdot \;10^{-4}$	-
Field uniformity in the good field region $\frac{\Delta B}{B}$ (23)	$\pm 4\;\cdot \;10^{-4}$	-

**Table 2:** Integrated normal field error harmonics and field uniformity, corresponding to the curves plotted in Figure 9. The harmonics are numbered according to the convention n = 2 quadrupole, n = 3 sextupole, etc., refer to the radius $r_{0}=$ 80 mm, and are normalized with respect to the integrated dipole field at each respective current level.

Current	*b*_2_	*b*_3_	*b*_4_	*b*_5_	$\frac{\Delta B}{B}$ (23)	Unit
1000 A	1.00	4.28	−1.87	−3.47	3.4	$10^{-4}$
3000 A	−0.02	3.90	1.70	−3.34	2.5	$10^{-4}$
5400 A	−0.77	1.89	1.68	−1.06	3.0	$10^{-4}$

**Table 3:** Noise content η (24) for the fixed coil and the ±30 mm pulsed SSW. The quoted uncertainty is the standard deviation over three consecutive repetitions.

Sampling Rate	η Fixed Coil	η Pulsed SSW	η Pulsed SSW	Unit
	**(1 MS/s Decimated)**	**(1 MS/s Decimated)**	**(Directly Sampled)**	
10 kS/s	5.8 ± 2.6	58 ± 11	56 ± 11	$10^{-6}$
100 kS/s	3.7 ± 2.0	17 ± 3.5	17 ± 3.5	$10^{-6}$
250 kS/s	3.6 ± 1.7	10 ± 1.7	9.6 ± 1.7	$10^{-6}$
500 kS/s	3.6 ± 1.7	5.0 ± 2.0	4.9 ± 1.4	$10^{-6}$
1 MS/s	3.6 ± 1.7	4.7 ± 1.4	4.7 ± 1.4	$10^{-6}$

**Table 4:** Dynamic performance of the p-SSW and fixed coil setups. Values were calculated from the results across 81 measurements. Relative values are presented with respect to the magnetic field at 5400 A (1.835 Tm).

Measurement Method	Current (A)	τ ( ms) Avg. ±σ	$A_{0}$ (10^−4^) Avg. ±σ	Fit Error (10^−4^) RMS
Fixed Coil	500	48 ± 17	3.0 ± 0.2	0.2
Fixed Coil	1000	93 ± 15	4.4 ± 0.3	0.2
Fixed Coil	1500	121 ± 14	4.9 ± 0.2	0.2
Fixed Coil	2000	85 ± 18	5.1 ± 0.2	0.2
Fixed Coil	2500	99 ± 11	5.5 ± 0.3	0.2
Fixed Coil	3000	116 ± 7	6.2 ± 0.2	0.2
Fixed Coil	4080	125 ± 7	6.6 ± 0.3	0.2
Fixed Coil	4700	142 ± 3	7.1 ± 0.2	0.2
Fixed Coil	5400	146 ± 3	14.9 ± 0.3	0.2
±5 mm p-SSW	5400	137 ± 42	15.2 ± 1.4	0.8
±10 mm p-SSW	5400	143 ± 21	14.7 ± 0.9	0.4
±20 mm p-SSW	5400	149 ± 14	14.6 ± 0.6	0.3
±30 mm p-SSW	5400	144 ± 9	14.5 ± 0.4	0.3

## Data Availability

The datasets presented in this article are not readily available because of time limitations. Requests to access the datasets should be directed to the corresponding author.
